# Risk Factors and Outcomes of Children with Congenital Heart Disease on Extracorporeal Membrane Oxygenation—A Ten-Year Single-Center Report

**DOI:** 10.3390/life13071582

**Published:** 2023-07-19

**Authors:** Antonio Amodeo, Milena Stojanovic, Tugba Erdil, Hitendu Dave, Robert Cesnjevar, Sebastian Paal, Oliver Kretschmar, Martin Schweiger

**Affiliations:** 1 Pediatric Cardiovascular Surgery, Pediatric Heart Center, Department of Surgery, University Children’s Hospital Zurich, 8032 Zurich, Switzerlandhitendu.dave@kispi.uzh.ch (H.D.); robert.cesnjevar@kispi.uzh.ch (R.C.);; 2 Children’s Research Center, University Children’s Hospital Zurich, 8032 Zurich, Switzerland; 3 Division of General Thoracic Surgery, Inselspital, Bern University Hospital, University of Bern, 3010 Bern, Switzerland; 4 Pediatric Cardiology, Pediatric Heart Center, University Children’s Hospital Zurich, 8032 Zurich, Switzerland

**Keywords:** complications, congenital heart disease, ECMO, extracorporeal life support, mortality, risk factors, single ventricle

## Abstract

For children born with congenital heart defects (CHDs), extracorporeal life support may be necessary. This retrospective single-center study aimed to investigate the outcomes of children with CHDs on extracorporeal membrane oxygenation (ECMO), focusing on various risk factors. Among the 88 patients, 36 (41%) had a single-ventricle heart defect, while 52 (59%) had a biventricular defect. In total, 25 (28%) survived, with 7 (8%) in the first group and 18 (20%) in the latter. A *p*-value of 0.19 indicated no significant difference in survival rates. Children with biventricular hearts had shorter ECMO durations but longer stays in the intensive care unit. The overall rate of complications on ECMO was higher in children with a single ventricle (odds ratio [OR] 1.57, 95% confidence interval [CI] 0.67–3.7); bleeding was the most common complication in both groups. The occurrence of a second ECMO run was more frequent in patients with a single ventricle (22% vs. 9.6%). ECMO can be effective for children with congenital heart defects, including single-ventricle patients. Bleeding remains a serious complication associated with worse outcomes. Patients requiring a second ECMO run within 30 days have lower survival rates.

## 1. Introduction

Extracorporeal membrane oxygenation (ECMO) is a well-established therapy utilized in neonates and children. According to the Extracorporeal Life Support Organization (ELSO), the survival rates for patients undergoing ECMO treatment vary depending on the age group and the underlying condition. The survival rate for pediatric patients receiving extracorporeal cardiopulmonary resuscitation (eCPR) is 55%, while the survival rate for neonates receiving ECMO for respiratory problems is 84% [1].

Congenital heart defects (CHDs) are one of many factors that can affect how well children and newborns respond to ECMO. Neonatal children with CHDs have a reported 40% survival rate, compared to 48% for pediatric patients [1]. Patients with single-ventricle physiology within the CHD group are worse than those with biventricular CHDs in terms of outcomes [2]. Studies show that the survival of children in this subgroup is still low, with best-case survival rates being around 40% [3,4,5,6].

This study’s goal is to give a thorough overview of the particular population of CHD-affected children who need ECMO support. Our analysis aims to determine the risk factors and issues associated with ECMO use in these patients as well as the overall success of ECMO implantation. By examining all of these factors, just as other research groups are doing [7,8,9], we hope to advance patient care in this population and increase our understanding of CHD management in conjunction with ECMO therapy.

## 2. Materials and Methods

### 2.1. Study Design and Patient Population

This study is a retrospective, single-center investigation that aimed to evaluate the outcomes of children diagnosed with CHDs who required ECMO support between the years of 2009 and 2019. The study evaluated children up to the age of 16 and categorized them as either “neonates” if they were up to 30 days old or “pediatric” if they exceeded that age. To ensure the accuracy of the data, exclusion criteria were applied and consisted of excluding patients without a CHD diagnosis and those whose baseline or follow-up data were missing. Notably, patients who underwent a second ECMO run during the same hospital admission were considered separately and labeled as “second ECMO run”, with no patient receiving more than two ECMO runs.

### 2.2. Categorization of Patients Based on CHD Anatomical Classification

The patients were categorized into two groups based on the underlying anatomical classification of their CHD: those with biventricular CHD and those with single-ventricle CHD. The functional single-ventricle heart diagnosis encompassed conditions such as hypoplastic left heart syndrome, tricuspid valve atresia, pulmonary atresia, and borderline left ventricle.

### 2.3. Factors Leading to ECMO

This study identified five main categories of factors leading to the need for ECMO support: respiratory failure, cardiac failure, sepsis, post-cardiotomy ECMO, and eCPR (Table S1). The failure to wean off cardio-pulmonary bypass (CPB) or the need for ECMO within the first 48 h after a CPB-assisted operation were both referred to as post-cardiotomy ECMO.

Due to the complexity of patient cases, it was often challenging to attribute ECMO support to a single factor, as multiple relevant factors were concurrently present, influencing the decision to initiate ECMO support. In instances where multiple factors were identified, the patient was classified into the categories considered more relevant by two independent senior consultants. Any discrepancies were resolved through discussion until a consensus was reached. Consequently, for the majority of patients, it was established that more than one factor contributed to the need for ECMO support.

### 2.4. Complications and Criteria for Classifying Complications

A series of complications was defined and encompassed various situations. These included bleeding, reposition of a cannula, disseminated intravascular coagulation (DIC), circuit changes, and cerebrovascular insults (CVIs).

The main criterion for classifying bleeding as a complication was the need to repeat surgery due to bleeding. Any occurrence of bleeding, whether inside or outside the area of the index operation, was taken into account. Bleeding specifically related to ECMO was also considered. Regarding the repositioning of a cannula, such adjustments may be necessary either to improve ECMO flow or to correct unintentional displacement, and they were labeled as complications. The cleaning of cannulas or tubes to address thrombus formation also falls into this category of complications. Disseminated intravascular coagulation was evaluated based on laboratory and clinical assessments, constituting another complication category. This study also accounted for circuit changes, which were defined as changes resulting from the depletion of the oxygenator. In the study, CVI was defined as any thromboembolic event causing a cerebral insult during ECMO, confirmed through magnetic resonance imaging (MRI) or computed tomography (CT). These imaging modalities were interpreted by pediatric radiologists or neuroradiologists, ensuring the accurate identification and confirmation of CVI cases.

### 2.5. Non-Cardiac Genetic Diseases

In addition to the primary heart defect, some patients also had additional non-cardiac genetic diseases, which were documented separately and listed as “other diagnoses” (Table S2).

### 2.6. Data Storage and Ethics Approval

All data were safely stored in an Excel sheet protected by a password. The document containing personalized information linked to patient IDs was kept separate from the Excel sheet. The study received ethical approval from the Cantonal Ethics Committee Zurich (BASEC-Nr. 2020-01147).

### 2.7. Statistical Analysis

Data pertaining to patients and ECMO were gathered from the clinical information system “Phoenix^®^” provided by CGM Clinical (CompuGroup Medical Schweiz AG, Bern, Switzerland) at the University Children’s Hospital Zurich. A comprehensive set of variables was selected for analysis, including date of birth, gender, weight, classification as neonate or pediatric, presence of single-ventricle or biventricular heart, specific congenital heart defect, concomitant diseases, factors leading to ECMO initiation, occurrence of cardiopulmonary resuscitation (CPR) prior to ECMO, previous cardiotomy, last recorded pH and lactate levels before ECMO, type of ECMO implantation, cannulation site, timing of ECMO initiation and removal, occurrence of a second ECMO run, complications experienced while on ECMO, conversion to a ventricular assist device (VAD), length of stay in the intensive care unit (ICU), survival while on ECMO, and survival after a 1-year follow-up. To ensure the accuracy and consistency of the collected data, an Excel sheet was utilized for data collection. Subsequent statistical analysis and evaluation were conducted using the software “R-Studio” [RStudio Team (2020). RStudio: Integrated Development for R. RStudio, PBC, Boston, MA, USA]. Results are given as mean values ± standard deviation or numbers and percentages. Comparisons were performed using Student’s *t*-test or the Mann–Whitney *U*-test. Qualitative variables were analyzed using Fisher’s exact test. The survival times of the two groups were compared using a log-rank test, with statistical significance assumed at a 2-sided *p*-value of 0.05 or lower.

## 3. Results

### 3.1. Study Group

A total of 88 patients received ECMO therapy, including 42 neonates and 43 females. The mean age at ECMO implantation was 1.4 years, with a standard deviation of 3.6 years. The average weight of the patients was 6.9 kg, with a standard deviation of 1.0 kg. Table 1 shows the specific baseline characteristics for both groups.

Single-ventricle heart defects were found in 36 (41%) of the 88 patients, with hypoplastic left heart syndrome (HLHS) being the most prevalent condition in this cohort, accounting for 23 cases (26%). As shown in Table 2, which displays the distribution of CHD types across the sample, 52 patients (59%) had a congenital heart defect with biventricular physiology.

In both the single-ventricle and biventricular groups, cardiac failure emerged as the main cause of ECMO. Cardiovascular failure was identified in 33 of the 36 patients with a single-ventricle defect (91%) and 48 patients (92%), respectively, in the biventricular group. Notably, the majority of single-ventricle patients (70%) required ECMO support post-cardiotomy, compared to 55% of the biventricular patients. Respiratory failure was a contributing factor in 44% of single-ventricle cases and in 32% of biventricular cases. Sepsis, on the other hand, was a relatively uncommon cause of ECMO, accounting for 5% of cases in the single-ventricle group and 2% in the biventricular group.

Table 3 provides further information on the ECMO-related data for the two groups.

### 3.2. Outcomes

**Figure 1:**
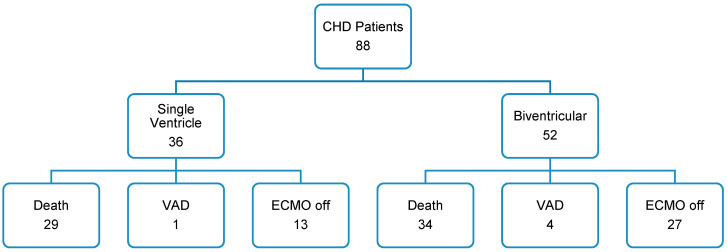
Outcome: this chart shows the number of patients in absolute numbers.

**Figure 2:**
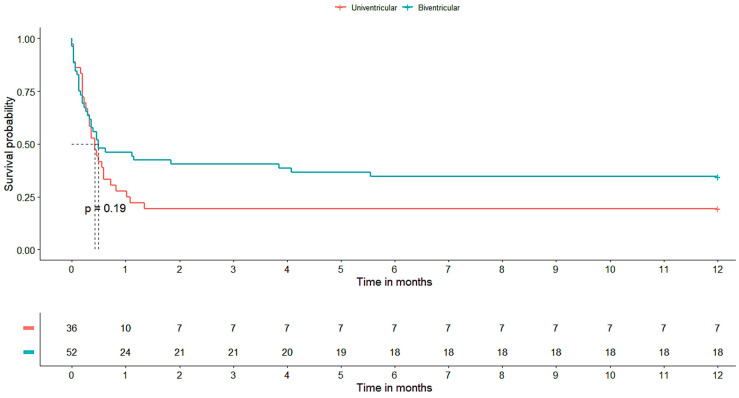
Survival after ECMO. The Kaplan–Meier curve shows the survival time of patients from ECMO implantation to one-year follow-up. The risk table shows survival in absolute numbers.

The overall survival rate for children with CHDs receiving ECMO support was found to be poor. Out of the 88 patients included in the study, 54% did not survive while on ECMO support. However, 45% of the patients were successfully weaned off ECMO. Additionally, 6% of the patients required VAD support, and 28% of the patients were discharged from the hospital. Figure 1 presents a schematic representation of the main outcomes in absolute numbers:

At the time of hospital discharge and one year after, 28% of the patients included in the study were still alive. In total, 7 (28%) of these survivors had a single-ventricular heart, whereas the remaining 18 (72%) had biventricular physiology. Notably, whether univentricular or biventricular, the type of underlying cardiac disease did not show a significant impact on survival outcomes (*p* = 0.19). The survival curve corresponding to these findings is visually represented in Figure 2, providing a graphical representation of the observed survival rates over time.

The average duration of ECMO support was 5.4 days, with a standard deviation of 4.4 days. Compared to children with univentricular hearts, children with biventricular hearts had shorter ECMO support durations while experiencing longer stays in the intensive care unit (ICU). However, the difference in ICU stays between the two groups was not found to be statistically significant (*p*-value: 0.99), as indicated by the analysis. The outcome data are listed in Table 4.

It was found that ECMO-support-related complications were frequent, affecting about 49% of patients. Below and in Table 5 are descriptions of these issues in more detail. Importantly, patients with univentricular hearts showed significantly increased frequency of overall complications (odds ratio: 1.57, 95% confidence interval: 0.67–3.7). The most common complication seen in both groups was bleeding.

Among the 33 patients who underwent eCPR prior to ECMO implantation, 30% survived to hospital discharge and were still alive at one year. Prior to ECMO implantation, CPR lasted an average of 49 min for both groups. The mean time spent performing CPR was notably shorter, at 23 min, among the patients who survived.

### 3.3. Subgroup Second ECMO Run

**Figure 3:**
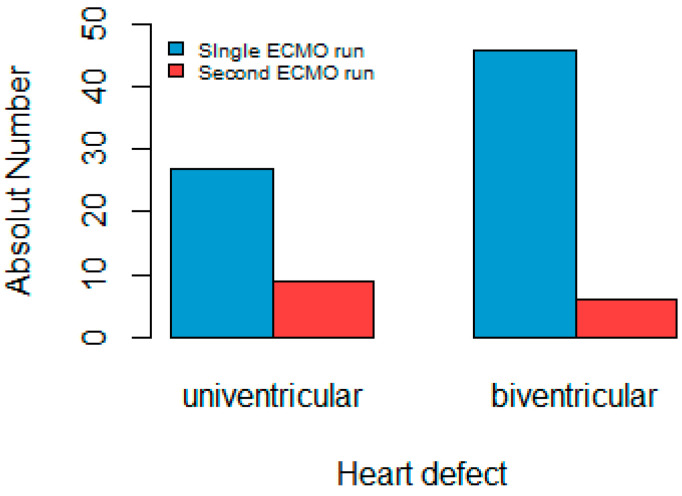
Second ECMO run. The graph shows that 8 children (9%) with univentricular heart defects and 5 children (6%) with biventricular heart defects required a second ECMO run.

An analysis was conducted specifically on the subset of patients who underwent a second ECMO run, which included a total of 13 individuals (15% of the total). Figure 3 illustrates that out of this group, the majority (eight patients) had a single ventricle. With seven cases, hypoplastic left heart syndrome was identified as the most common heart defect.

Cardiac failure was the main factor leading to ECMO in this group, accounting for 85% of cases, and the second ECMO run was started on average 1.9 days after the first run. The average support duration for the children who needed a second ECMO run was 7.3 days, with a standard variation of 1.2 days. Only 2 of the 13 patients survived through the second ECMO run. These two survivors, who were pediatric patients, both had a single-ventricle heart abnormality.

Complications were observed in 46% of the cases within the subset of patients who underwent a second ECMO run. Among the patients with a univentricular heart defect, complications occurred in four children, representing 50% of this subgroup. Similarly, among the patients with a biventricular heart defect, complications were observed in two individuals, accounting for 40% of this subgroup. Within this cohort, reoperation due to bleeding emerged as the most frequent complication. It is worth highlighting that the two children who survived the second ECMO run did not experience any complications while on ECMO support. Additionally, none of the children in this group required the implantation of a ventricular assist device.

## 4. Discussion

The use of ECMO in patients with congenital heart disease (CHD) is increasing globally [10,11,12,13,14]. In our study, we found that cardiac failure, not surprisingly, was the most common factor leading to the need for ECMO, followed by post-cardiotomy failure. Almost half of the patients (45%) were successfully weaned from ECMO, while a small but substantial number of patients (*n* = 6; 7%) required long-term mechanical circulatory support in the form of a ventricular assist device. For those patients who were successfully discharged, the survival rate at one year was consistent with the rate at hospital discharge, with a survival rate of 22% for neonates and 35% for pediatric patients. When we examined the underlying anatomy of the total ECMO cohort, we found that a substantial number (41%) of the patients had single-ventricle hearts. The survival rate for children on ECMO with biventricular heart abnormalities is approximately 40%, and the survival rate for children with single-ventricle hearts is lower [15,16,17].

The wide range of survival rates reported In the literature is noteworthy and can be attributed to differences in inclusion and exclusion criteria, center size, study dimension (single-center vs. multicenter), and the timing and setting of ECMO deployment (daytime vs. nighttime), even though it has been found that the latter variable was irrelevant in adults [18] and in pediatric patients [19]. Most of the studies found in the literature primarily included children who underwent ECMO after cardiac surgery [12,15,20]. In contrast, our study aimed to include all children who required ECMO, regardless of their correction status or specific heart defect, provided that ECMO was indicated and subsequently implemented. This approach allowed us to avoid selection bias. Our findings are consistent with the survival rate ranges found in the literature [6,15,21] with a survival rate of 35% for biventricular hearts and 19% for single-ventricle hearts. Importantly, we did not observe a significant difference in survival based on the underlying anatomy, whether the patient had a biventricular or univentricular defect. In the literature, there are conflicting reports, with some studies showing no significant difference in outcome as we found [15,22] or showing a better outcome for children with biventricular CHD, when ECMO is needed [16]. It should be noted that dividing patients based on underlying anatomy creates groups (biventricular and single-ventricle CHD) that inherently contain a heterogeneous patient population.

Factors other than anatomy may also impact survival probability. High inotrope scores, pre-ECMO acidosis, elevated pre-ECMO lactate, failure to clear lactate within 24 h, bleeding while on ECMO, fluid overload, peripheral cannulation, renal failure, start of ECMO in the intensive care unit, and length of ECMO support are some of the factors that have been discussed in the literature, although not always with consistent conclusions [23,24,25,26]. In terms of pH and lactate levels, we found higher lactate levels and lower mean pH values in the group of children with biventricular hearts (pH 7.06 vs. 7.23, lactate 6.93 vs. 6.05). Even so, this group’s likelihood of survival was better than that of children with single-ventricle hearts (35% vs. 19%).

Bleeding during ECMO is identified as a significant risk factor in the literature and is known to reduce the likelihood of survival [12,27,28]. The incidence of bleeding in cardiac patients on ECMO ranges from 25% to 78%, with children who have a structural heart defect being at an increased risk of bleeding [29,30,31,32]. Our study also demonstrated a high rate of bleeding complications during ECMO. However, the prevalence of reoperation due to bleeding did not show a significant difference between children with single-ventricle and biventricular hearts (25% vs. 29%). Nonetheless, the mortality rate for children with a single ventricle and this complication was 100%, compared to 80% for children with biventricular CHD.

Given the prevalence of post-cardiotomy cases or failures to wean from cardiopulmonary bypass as reasons for ECMO, the majority of patients in our sample had central cannulation. It is possible that the slightly higher survival rate (27.3% vs. 22.2%) seen in patients with central cannulation can be attributed to the use of larger cannulas and increased flow [33]. The benefits of central cannulation in post-cardiotomy adult patients are being discussed, and contrasting findings have been found in the literature [34,35]. Peripheral ECMO may have the benefit of a lesser risk of bleeding [36] but pose a risk of limb ischemia [37].

Another risk factor can be identified in the presence of genetic abnormalities other than the ones strictly related to CHD. In children with a congenital heart defect, the incidence of non-cardiac and genetic illnesses ranges from 15 to 30% [38,39], which is consistent with the findings in our cohort (24%). According to the abovementioned study by Alsoufi et al., genetic abnormalities are a significant risk factor for mortality. We also observed that children with non-cardiac genetic illnesses had a lower survival rate than those without them (9.5% vs. 26.1%).

Mortality after eCPR is still high, and longer CPR duration correlates with higher mortality [40,41,42]. In our study population, 38% of children with ECMO received eCPR. Among these, 10 patients (11%) were discharged from hospital and are still alive after one year. It is worth noting that eight patients (9%) had a biventricular circulation, compared to two patients (2.3%) who had a univentricular heart. In our analysis, the mean duration of CPR prior to the implantation of an ECMO device was 49 min; the 10 survivors received CPR for an average of 23 min. We did not look into the neurological outcomes of these patients, but one objective of future studies should be examining the long-term effects of such issues. Several investigations [40,43,44] have shown that neurological complications are frequent in this patient subgroup.

In our analysis, the survival of children who required a second ECMO run was worse compared to those who had only one run (15% vs. 28% survival). Following a second ECMO run, the literature indicates survival rates of about 25% [45,46,47,48]. In our cohort, 13 patients (15%) required a second ECMO run, of whom around half (*n* = 6) were neonates. The outcomes were poor, with only two children surviving the second ECMO run. Both survivors were pediatric patients with hypoplastic left heart syndrome. Although the survival results were lower with the second ECMO run, there were no significant differences in the incidence of complications (45.9% vs. 46.1%).

### Limitations

Several limitations should be considered in the interpretation of our findings, given that this study was retrospective and conducted at a single center. The relatively small sample size of 88 patients and the long observation period of 10 years may have implications for the generalizability of the results.

It is important to point out that the decision-making process was frequently influenced by numerous factors rather than a single determinant, and that the indication for ECMO implantation was determined retrospectively based on the data that were available.

We acknowledge that we did not report the ultimate cause of death for the patients in our cohort. While failure of cardiac function recovery could be attributed as a cause of death, it is essential to recognize that there may be other contributing factors, such as sepsis, intracranial hemorrhage, or multi-organ failure, which could have played a significant role. Future studies should aim to provide a more comprehensive analysis of the ultimate causes of mortality in this patient population.

It is important to highlight that our study did not conduct statistical analysis to determine whether there were significant differences between the subgroups of patients who underwent eCPR and those who did not receive CPR, instead choosing to focus on descriptive representation. We were unable to conduct thorough statistical analysis for this particular comparison due to insufficient data (unfortunately, CPR duration data were only available for 18 of the 23 non-survivors). Therefore, our findings regarding the differences between these two variables should be interpreted cautiously. Further research is necessary to identify any appreciable differences in survival or other outcomes between patients who received eCPR and those who did not in order to better understand the features and outcomes of these subgroups.

## 5. Conclusions

Our findings suggest that neonates and children with congenital heart defects can be successfully treated with ECMO, even in the presence of single-ventricle heart defects. Bleeding, however, continues to be a serious complication linked to worse outcomes. Patients who require a second ECMO run within 30 days continue to have a low survival rate. In order to address the study’s limitations and examine options for enhancing results in this patient population, additional research is necessary.

## Figures and Tables

**Table 1:** Baseline characteristics.

	Single Ventricle		Biventricular	
n	36		52	
Age at ECMO implantation in months (mean (±SD))	28	(54.7)	9.9	(31.6)
Male (%)	16	(18)	29	(33)
Female (%)	20	(23)	23	(26)
Neonate (%)	13	(15)	29	(33)
Paediatric (%)	23	(26)	23	(26)
Other diagnosis (%)	5	(6)	16	(18)

**Table 2:** Congenital heart defects.

	Total Cases (n = 88)	
Cardiac Diagnosis	n	%
Single ventricle	36	41
Functional single ventricle (non-HLHS)	13	15
HLHS	23	26
Biventricular	52	59
VSD	24	27
ASD	18	20
AVSD	19	22
TOF	5	6
TGA	14	16
Aortic valve stenosis	11	13
Truncus arteriosus communis	4	5
Pulmonary valve stenosis	3	3
Pulmonary atresia	13	15
IAA	3	3
TAPVD	9	10
Partial anomalous pulmonary venous connection	2	2
DORV	2	2
ALCAPA	3	3
Atrioventricular valve regurgitation	15	17
Myocarditis	1	1
Cardiomyopathy	3	3

**Table 3:** ECMO Data.

	Single Ventricle		Biventricular	
n	36		52	
CPR time in minutes (mean (±SD))	49.44	(40.44)	49.44	(52.78)
Surgery pre-ECMO (%)	33	(37.5)	36	(41)
ECMO pre-surgery (%)	3	(3.4)	11	(12.5)
pH pre-ECMO (mean (±SD))	7.23	(0.15)	7.06	(0.66)
Lactate pre-ECMO (mean (±SD))	6.05	(4.1)	6.93	(5.59)
VA-ECMO (%)	33	(37.5)	48	(54)
VV-ECMO (%)	3	(3.4)	4	(4.5)
Central cannulation (%)	33	(37.5)	44	(50)

**Table 4:** Outcome: single ventricle versus biventricular.

	Single Ventricle		Biventricular	
n	36		52	
ECMO duration in days (mean (±SD))	5.9	(4.7)	5	(4.3)
ICU stay in days (mean (±SD))	16	(14)	23	(34)
Second ECMO run (%)	8	(9)	5	(6)
Change to VAD (%)	1	(1.1)	4	(4.5)

**Table 5:** Complications of ECMO.

	Single Ventricle		Biventricular	
n	36		52	
Reoperation due to bleeding (%)	9	(10)	15	(17)
Reposition of cannula (%)	8	(9)	11	(12.5)
Cleaning the cannula due to a thrombus (%)	1	(1.1)	0	(-)
DIC (%)	6	(7)	3	(3.4)
Verified CVI (%)	5	(5.6)	2	(2.2)
Circuit change (%)	2	(2.2)	4	(4.5)

## Data Availability

All of the data of this study are in the Hospital Database, available with the software “Phoenix^®^” of CGM Clinical (CompuGroup Medical Schweiz AG, Bern, Switzerland).

## Supplementary Materials

The following supporting information can be downloaded at: https://www.mdpi.com/article/10.3390/life13071582/s1, Table S1: Factors leading to ECMO implantation; Table S2: other diagnosis.

## Abbreviations

Abbreviation	Meaning
ALCAPA	anomalous left coronary artery from the pulmonary artery
ASD	atrial septal defect
AVSD	atrioventricular septal defect
CBP	cardiopulmonary bypass
CT	computer tomography
CVI	cerebrovascular insult
CHD	congenital heart disease
DORV	double outlet right ventricle
DIC	disseminated intravascular coagulation
eCPR	extracorporeal cardiopulmonary resuscitation
ECMO	extracorporeal membrane oxygenation
ELSO	extracorporeal life support organization
HLHS	hypoplastic left heart syndrome
IAA	interrupted aortic arch
ICU	intensive care unit
MRI	magnetic resonance imaging
TAPVD	total anomalous pulmonary venous drainage
TGA	transposition of the great arteries
TOF	tetralogy of Fallot
VA	venoarterial
VAD	ventricular assist device
VSD	ventricular septum defect
VV	venovenous
