# Supplementary material for: Risk Factors and Outcomes of Children with Congenital Heart Disease on Extracorporeal Membrane Oxygenation—A Ten-Year Single-Center Report
Source: Life (Basel). 2023 Jul 19;13(7):1582. doi: 10.3390/life13071582 (PMC10381661; doi:10.3390/life13071582)
Supplement: Supplementary file 1 [file life-13-01582-s001.zip › life-2502666-supplementary.pdf]

Table S1: Factors leading to ECMO Implantation.

Category	
Respiratory problem / lung failure	❖ ARDS<br>❖ Pneumonia (viral/bacterial/aspiration)<br>❖ Pulmonary hypertension
Cardiac failure	❖ Underlying cardiac diagnosis<br>❖ Intractable arrhythmia<br>❖ Myocarditis
Sepsis	❖ Hospital-acquired infections (postoperative infection, catheter-associated infection)<br>❖ Out of hospital-acquired infections
Postcardiotomy ECMO	❖ Failure to wean from CPB<br>❖ Stunned myocardium<br>❖ Low cardiac output syndrome
eCPR	❖ Cardiac arrest refractory to CPR

ARDS - acute respiratory distress syndrome; CPB - cardiopulmonary bypass; ECMO - extracorporeal membrane oxygenation; eCPR - extracorporeal cardiopulmonary resuscitation.

Table S2: Other Diagnosis

- Asplenia
- Cardiac tumor
- Congenital hypothyroidism
- Cornelia-de-Lange-syndrome
- Cystic fibrosis
- DiGeorgie syndrome
- Hypogammaglobulinemia
- Kartagener syndrome
- Macrocephaly
- Situs inversus abdominalis
- Trisomy 21
- Uhl's disease
- Ulrich-Turner syndrome
- VACTERL association
